# The leukodystrophy mutation *Polr3b* R103H causes homozygote mouse embryonic lethality and impairs RNA polymerase III biogenesis

**DOI:** 10.1186/s13041-019-0479-7

**Published:** 2019-06-20

**Authors:** Karine Choquet, Maxime Pinard, Sharon Yang, Robyn D. Moir, Christian Poitras, Marie-Josée Dicaire, Nicolas Sgarioto, Roxanne Larivière, Claudia L. Kleinman, Ian M. Willis, Marie-Soleil Gauthier, Benoit Coulombe, Bernard Brais

**Affiliations:** 1 Montreal Neurological Institute, McGill University, 3801 University Street, room 622, Montréal, Québec, H3A 2B4 Canada; 2 Department of Human Genetics, McGill University, Québec, Montréal Canada; 3 Lady Davis Institute for Medical Research, Jewish General Hospital, Québec, Montréal Canada; 4 Translational Proteomics Laboratory, Institut de recherches cliniques de Montréal (IRCM), Québec, Montréal Canada; 5 Department of Biochemistry, Albert Einstein College of Medicine, New York, Bronx USA; 6 Département de biochimie et médecine moléculaire, Université de Montréal, Québec, Montréal Canada

**Keywords:** Leukodystrophy, RNA polymerase III, Mouse model, *POLR3A*, *POLR3B*, Myelination

## Abstract

Recessive mutations in the ubiquitously expressed *POLR3A* and *POLR3B* genes are the most common cause of POLR3-related hypomyelinating leukodystrophy (POLR3-HLD), a rare childhood-onset disorder characterized by deficient cerebral myelin formation and cerebellar atrophy. *POLR3A* and *POLR3B* encode the two catalytic subunits of RNA Polymerase III (Pol III), which synthesizes numerous small non-coding RNAs. We recently reported that mice homozygous for the *Polr3a* mutation c.2015G > A (p.Gly672Glu) have no neurological abnormalities and thus do not recapitulate the human POLR3-HLD phenotype. To determine if other POLR3-HLD mutations can cause a leukodystrophy phenotype in mouse, we characterized mice carrying the *Polr3b* mutation c.308G > A (p.Arg103His). Surprisingly, homozygosity for this mutation was embryonically lethal with only wild-type and heterozygous animals detected at embryonic day 9.5. Using proteomics in a human cell line, we found that the POLR3B R103H mutation severely impairs assembly of the Pol III complex. We next generated *Polr3a*^*G672E/G672E*^*/Polr3b*^*+/R103H*^double mutant mice but observed that this additional mutation was insufficient to elicit a neurological or transcriptional phenotype. Taken together with our previous study on *Polr3a* G672E mice, our results indicate that missense mutations in *Polr3a* and *Polr3b* can variably impair mouse development and Pol III function. Developing a proper model of POLR3-HLD is crucial to gain insights into the pathophysiological mechanisms involved in this devastating neurodegenerative disease.

**Electronic supplementary material:**

The online version of this article (10.1186/s13041-019-0479-7) contains supplementary material, which is available to authorized users.

## Background

Recessive mutations in *POLR3A*, *POLR3B, POLR1C* and *POLR3K* cause RNA Polymerase III-related hypomyelinating leukodystrophy (POLR3-HLD), [1–4] a devastating childhood-onset neurodegenerative disorder characterized by motor regression, cerebellar features and/or cognitive dysfunction, as well as hypomyelination and cerebellar atrophy on magnetic resonance imaging (MRI) [5]. To date, more than 100 POLR3-HLD cases have been reported. The majority of these involve mutations that are present in a single family or in only a handful of patients [5]. There is significant phenotypic heterogeneity regarding severity, age of onset and nature of symptoms and no clear genotype-phenotype correlation has been observed [5]. *POLR3A* and *POLR3B* encode the two catalytic subunits of RNA Polymerase III (Pol III), an essential enzyme responsible for the synthesis of nuclear-encoded transfer RNAs (tRNA) and various other small housekeeping non-coding RNAs (ncRNA). However, none of these Pol III transcripts have a known role in myelination and/or cerebellar function, highlighting the need for a disease model in which the pathophysiological mechanisms responsible for POLR3-HLD can be investigated. We recently reported that mice homozygous for the French Canadian founder mutation *Polr3a* c.2015G > A (p.Gly672Glu) have normal motor function, myelination and cerebellar integrity and thus do not recapitulate the human POLR3-HLD phenotype [6]. Nonetheless, it remains unknown whether other mutations in *Polr3a* or *Polr3b* can lead to a POLR3-HLD phenotype in mice.

In this follow-up study, we assessed the impact of a different POLR3-HLD-causing mutation, *Polr3b* c.308G > A (p.Arg103His), and found that it has a severe impact on mouse development and on Pol III biogenesis, indicating that mutations in Pol III subunits have variable effects in mice that may be a consequence of their specific defects on Pol III function. Second, we show that increasing the Pol III mutational burden of *Polr3a* G672E mice by adding a heterozygous *Polr3b* mutation does not lead to significant impairment at the phenotypic, histological or transcriptional levels, indicating that one functional *Polr3b* allele is sufficient to maintain normal Pol III function even in the presence of two mutated *Polr3a* alleles, providing further evidence that the *Polr3a* G672E mutation mimics a wild-type allele in mouse and that vulnerability to Pol III mutations varies between species.

## Results and discussion

**Fig. 1:**
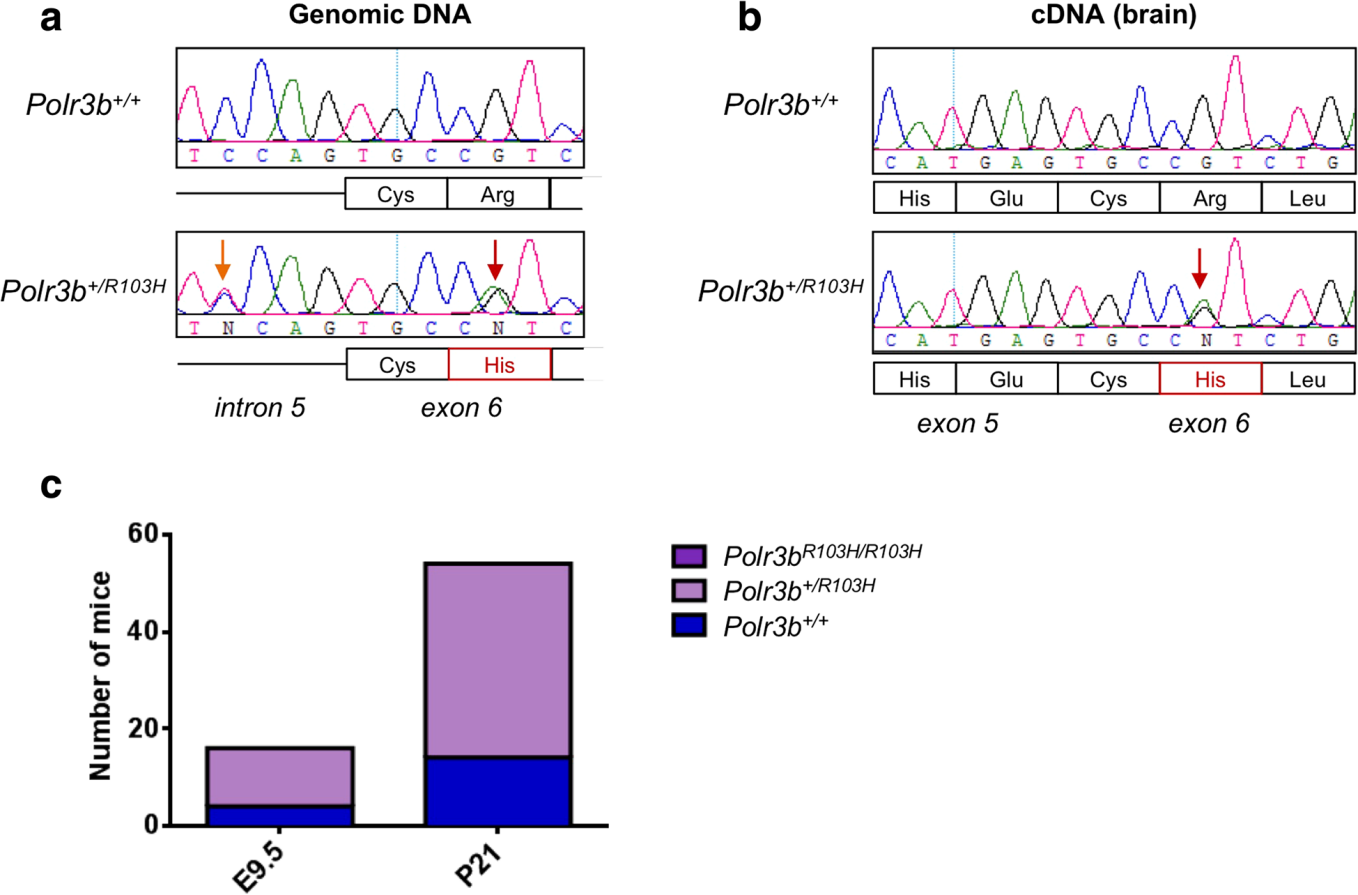
Generation of a *Polr3b* KI mouse model. **a** Genomic DNA chromatograms of *Polr3b* exon 6 in WT and *Polr3b*^*+/R103H*^ heterozygous mice. The *Polr3b* mutation c.308G > A (p.R103H) is indicated by a red arrow and the silent intronic mutation is indicated by an orange arrow. **b** cDNA chromatograms of *Polr3b* exons 5 and 6 in WT and *Polr3b*^*+/R103H*^ heterozygous mice. The *Polr3b* mutation c.308G > A (p.R103H) mutation is indicated by a red arrow. Both alleles are seen in the heterozygous mice, suggesting that the mutation does not cause abnormal splicing. **c** Homozygous mice for the *Polr3b* R103H mutation do not survive embryogenesis. Heterozygous parents were bred and E9.5 embryos or live pups were genotyped. No homozygous mice (*Polr3b*^*R103H/R103H*^) were observed in a total of 54 live pups and 16 embryos genotyped

To determine if other mutations in *Polr3a* or *Polr3b* cause a leukodystrophy phenotype in mice, we acquired heterozygous *Polr3b* c.308G > A (p.Arg103His) mice, which were generated using CRISPR-Cas9. Since this knock-in mouse was being produced in parallel with *Polr3b*^*−/−*^ mice as part of the NorCOMM2 project, the c.308G > A mutation was selected because of its proximity to the targeting site of the single guide RNA used to generate *Polr3b*^*−/−*^ mice. This mutation has only been observed in one POLR3-HLD patient in compound heterozygosity with *POLR3B* c.1568C > T (p.Val523Glu) [5]. We confirmed the presence of the heterozygous c.308G > A mutation in these animals by Sanger sequencing of tail genomic DNA (Fig. 1a). However, when we bred heterozygous mice together, we were unable to obtain homozygous *Polr3b*^*R103H/R103H*^ mice. After weaning, only wild-type (WT) (14/54) and heterozygote animals (40/54) were detected (Fig. 1c). A similar distribution of genotypes was seen at embryonic day 9.5 (Fig. 1c). These results indicate that the *Polr3b* R103H mutation is embryonically lethal at or before mid-gestation, similarly to *Polr3a*^−/−^null mice [6]. This is in stark contrast to homozygous *Polr3a*^*G672E/G672E*^ mice, which are viable and do not display any abnormalities at 12 months of age [6]. POLR3A and POLR3B protein sequences are highly conserved between human and mouse, with perfect conservation surrounding the POLR3A G672 and POLR3B R103 sites (multiple protein alignment by Clustal Omega) [6, 7]. Thus, the phenotypic differences between the two mouse models cannot be explained by differences in sequence conservation levels between species.

The c.308G > A mutation is located near the start of exon 6 and was introduced alongside an intronic mutation (c.304-4C > T) to prevent CRISPR-Cas9 re-editing of the repaired genomic DNA. To verify whether these mutations cause aberrant splicing leading to a null allele, we performed RT-PCR and Sanger sequencing of exons 4–8 of the *Polr3b* cDNA in heterozygous mice. Normal splicing was confirmed (Fig. 1b). Furthermore, both alleles were detected in similar proportions on the chromatogram, indicating that the mutant allele is not degraded. Thus, the c.308G > A mutation does not have a detrimental impact on mRNA splicing or stability. Taken together, results from *Polr3a*^G672E/G672E^ and *Polr3b*^+/R103H^ mice may suggest that human missense mutations in *Polr3a* and *Polr3b* can have no impact in mice or lead to early developmental arrest.

**Fig. 2:**
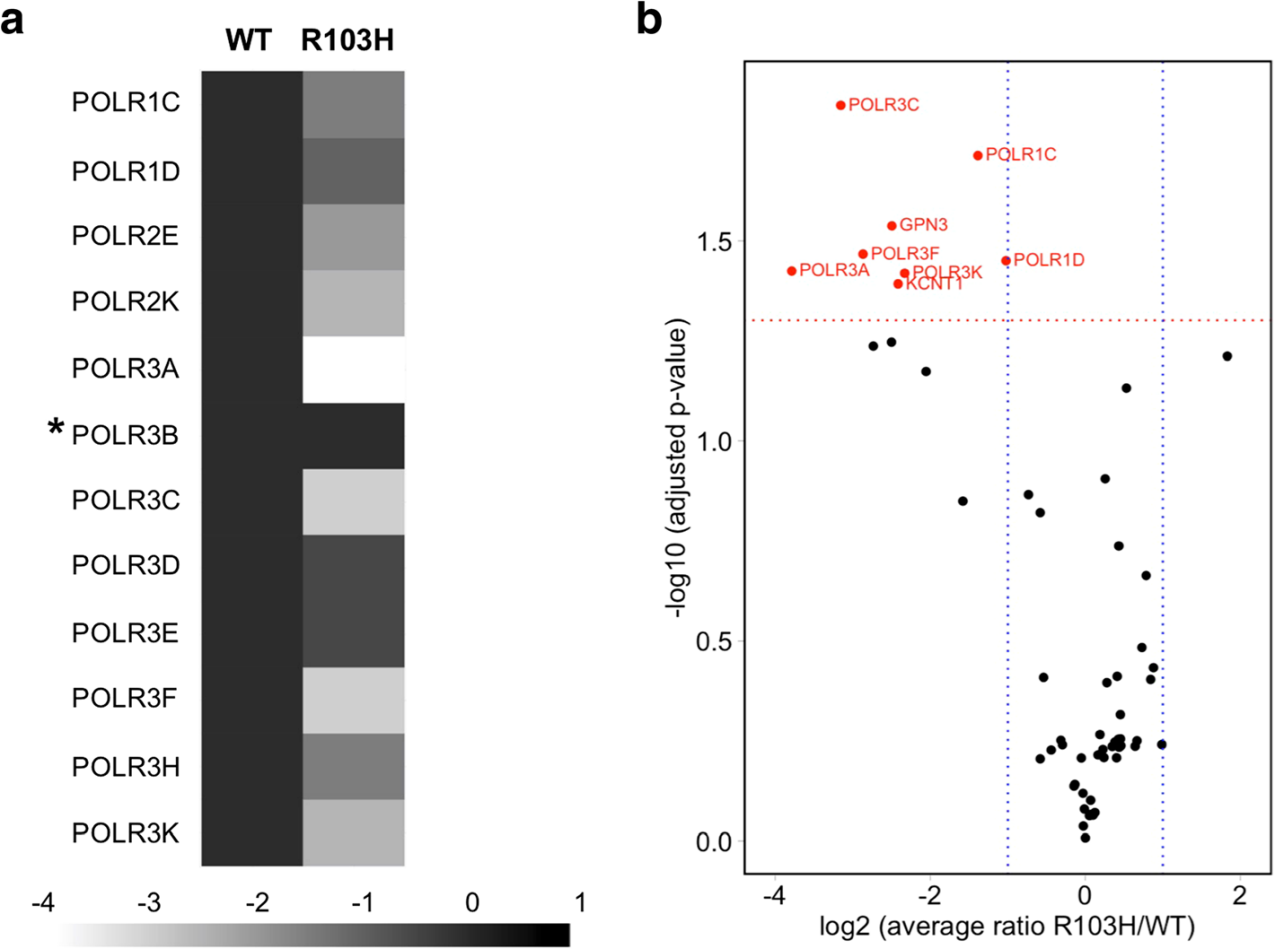
Impact of POLR3B R103H mutation on the assembly of Pol III complex**.** FLAG-tagged POLR3B WT or R103H were expressed in HEK293 cells, purified using an anti-FLAG antibody and digested with trypsin. The co-purified proteins were identified by LC-MS/MS mass spectrometry. The spectral counts of each peptide were computed using X-Tandem and Perseus. **a** The heatmap contains the average of the log_2−_transformed spectral count ratios across all 4 replicates. POLR3B (the bait) is identified by an asterisk. **b** Volcano plot of the log_2_-transformed average spectral count ratio R103H/WT (x-axis) and the –log_10_ q-value obtained by using a two-tailed T-test adjusted with a permutation-based multiple hypothesis testing and an s0 correction factor of 0.1 (y-axis). Proteins marked in red are considered statistically significant

We previously demonstrated that the POLR3A G672E mutation has a mild impact on Pol III function in human cells, since it does not impair Pol III complex assembly or nuclear import and only leads to a mild decrease in POLR3A occupancy at target loci [6]. Considering the severity of the *Polr3b* R103H mutation in mice, we hypothesized that this mutation would have a more detrimental effect on Pol III function. To test this hypothesis, FLAG-tagged versions of the wild-type (WT) or mutant (R103H) forms of POLR3B were transiently expressed in HEK293 cells. Whole cell extracts were affinity purified using an anti-FLAG antibody and analyzed by label-free shotgun proteomics (AP-MS). [3] FLAG-tagged WT and R103H POLR3B expression levels (Additional File 1: Figure S1A-B) and pulled-down levels (Additional File 2: Table S1) were similar and POLR3B interactors (prey proteins) levels were normalized by the pulled-down levels of the bait (POLR3B) in each purification. We observed that the POLR3B R103H mutation greatly impedes the assembly or stability of the Pol III complex. Indeed, eleven of the sixteen remaining Pol III subunits were detected in pulldowns that used tagged WT POLR3B whereas lower amounts of each of these subunits relative to WT were pulled down using POLR3B R103H (Fig. 2a, Additional File 2: Table S1). The ratio of six of these subunits showed a statistically significant reduction in the mutant purifications compared to that of the WT (Fig. 2b), implying a lower affinity of the mutant POLR3B subunit for its partners in vivo and a decreased abundance of the mutant Pol III complex. These data suggest that in homozygous mouse cells, the defective Pol III biogenesis conferred by the mutant POLR3B subunit may underlie the impaired mouse embryonic development phenotype. Importantly, when combined with our previous study [6], our data suggest that mutations causing a milder effect on Pol III function, such as POLR3A G672E, may not lead to a phenotype in mice, perhaps because of inter-species differences in myelination [6]. On the other hand, some mutations that affect Pol III complex assembly or nuclear import and that are generally seen as compound heterozygous alleles in humans may be too severe in the homozygous state, thus preventing embryonic development. Indeed, numerous POLR3-HLD cases with compound heterozygous alleles are known to include one allele with a frameshift or nonsense mutation [5]. Since we have only studied two mutations thus far, further experiments on other *POLR3A* and *POLR3B* mutations will be necessary to confirm such a correlation. This does not preclude that other mutations, perhaps ones that have a moderate effect on chromatin occupancy or transcription elongation, might lead to a clinical mouse phenotype recapitulating the human disease. We recently showed that in human cell lines, the POLR3-HLD-causing *POLR3A* M862 V mutation does not impair Pol III complex assembly or nuclear import but leads to decreased levels of a subset of Pol III transcripts, including tRNAs and 7SL RNA [8]. Thus, this mutation appears to have an intermediate functional phenotype in human cells that could perhaps lead to a neurological phenotype in mouse. Examining the functional impact of a larger panel of mutations in human cells could help pinpoint those that may produce a mouse phenotype, though mouse brain development may be less susceptible than the human brain to the impact of such mutations.

**Fig. 3:**
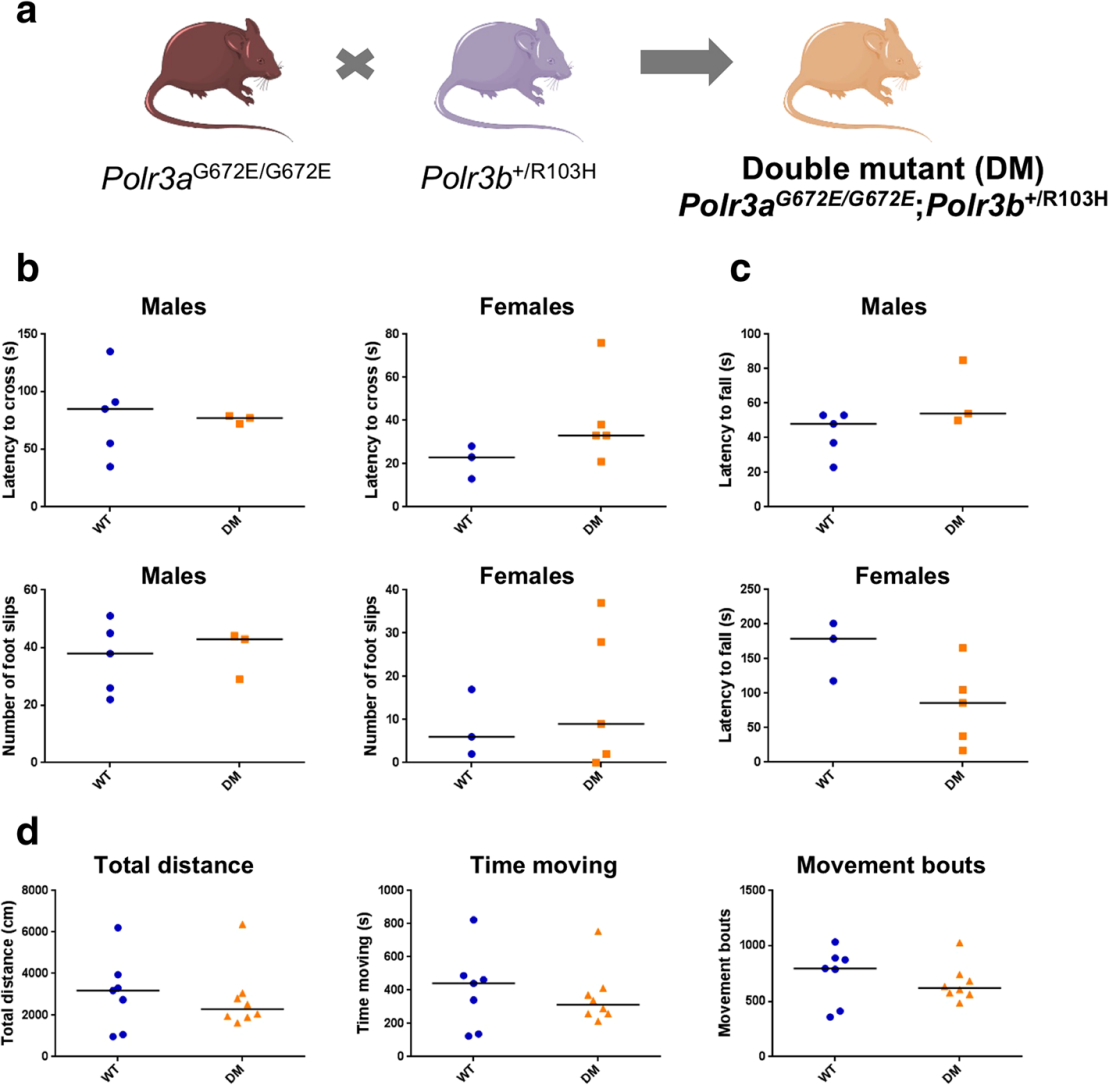
Normal motor function in double mutant mice. **a** *Polr3a*^*G672E/G672E*^ and *Polr3b*^*+/R103H*^ mice were crossed to generate double mutant mice that are homozygous for the *Polr3a* G672E mutation and heterozygous for the *Polr3b* R103H mutation. **b** Results from the 6 mm beam test at 6 months of age in males and females. Latencies to cross (top) and number of foot slips (bottom) are shown. For each mouse, three trials were performed and summed. **c** Results from the rotarod at 6 months of age in males and females. For each mouse, three trials were performed and summed. **d** Results from the open field test at 6 months of age in an independent cohort of males only. The open field test was run for 90 min per mouse during which total distance traveled (left), total time spent moving (middle) and number of movement bouts (right) were recorded for each 10 min interval

**Fig. 4:**
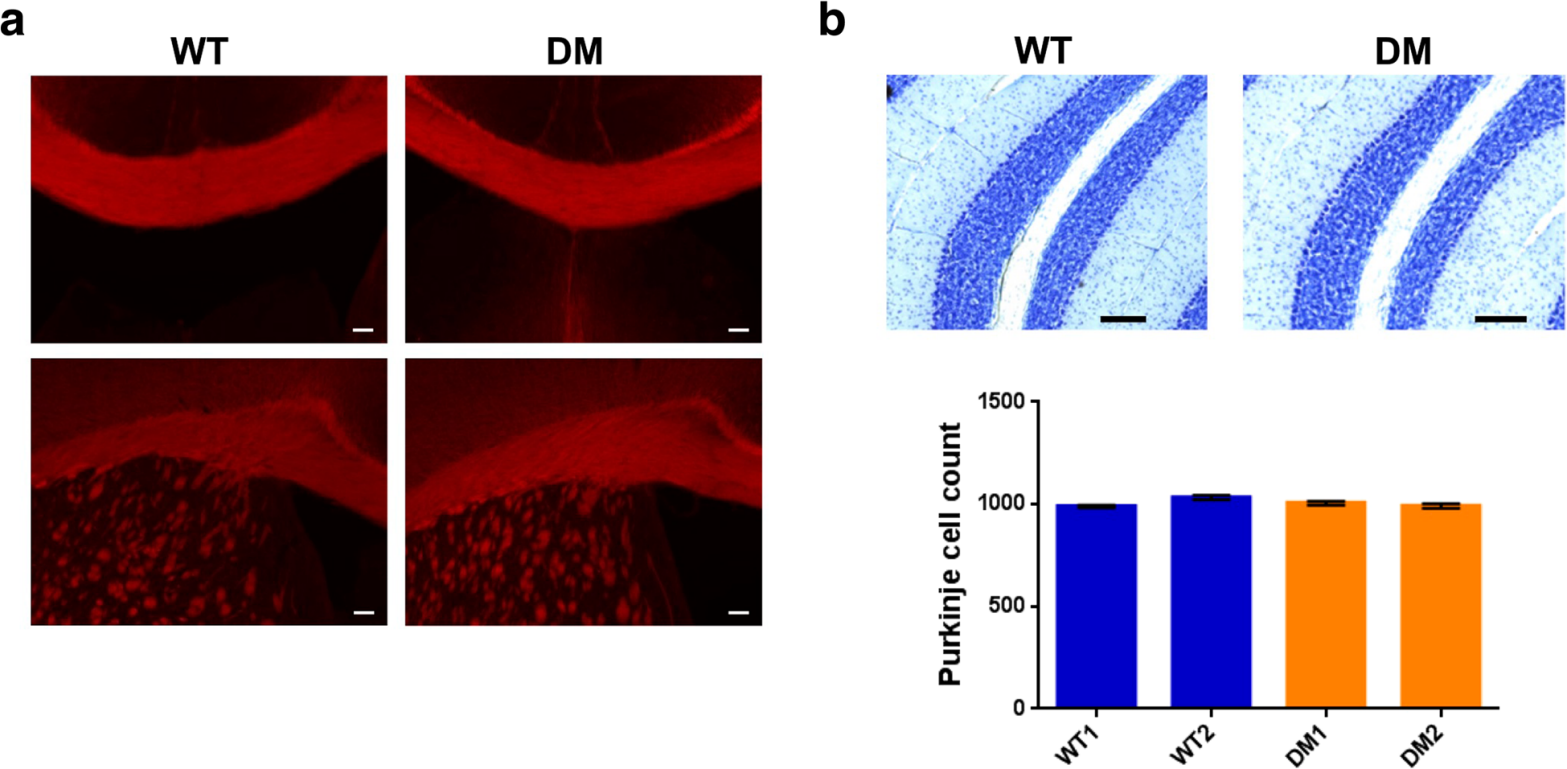
Normal myelination and cerebellar integrity in double mutant mice at 6 months of age. **a** Immunofluorescence for proteolipid protein (PLP) showing normal staining in the corpus callosum (top: coronal view; bottom: sagittal view) of DM mice compared to WT. Staining was performed on three mice per group and representative images are shown for each group. Scale bar: 100 μm. **b** Top: Nissl staining of sagittal cerebellar sections performed on two mice per group. Representative images are shown for each group. Scale bar: 100 μm. Bottom: Purkinje cell counts of mid-sagittal cerebellar sections. Data are represented as mean +/− SEM of three sections per mouse

**Fig. 5:**
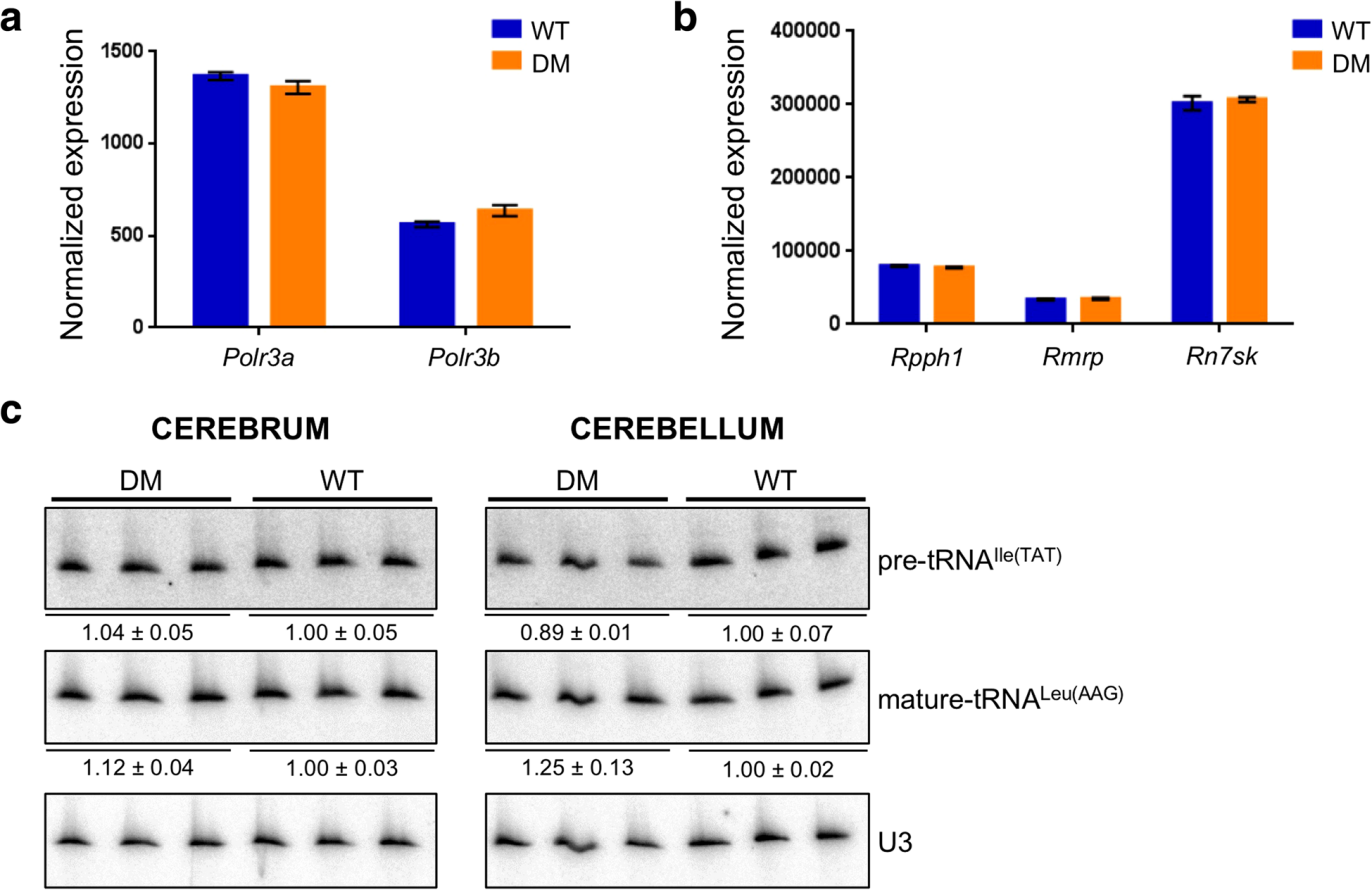
Normal expression of *Polr3a* and *Polr3b* mRNAs and Pol III transcripts in double mutant mice at 6 months of age**. a**, **b** Expression levels of **a**) *Polr3a* and *Polr3b* and **b**) three Pol III transcripts measured by RNA-seq in the cerebrum of three WT and three DM mice. Expression was normalized with DESeq2. Data are represented as mean +/− SEM. **c** Northern blots of precursor (pre) and mature tRNA species from the cerebrum (left) and cerebellum (right) of three WT and three DM mice. U3 snRNA, a Pol II transcript, was used as a loading control. Quantification of mean +/− SEM after normalization are indicated below the blots for each group

Considering the severe impact of the *Polr3b* R103H mutation on mouse development, we next asked whether introducing this mutation in the heterozygous state in *Polr3a*^*G672E/G672E*^ mice, thus increasing the Pol III mutational burden, could lead to a phenotype that would resemble POLR3-HLD. We crossed homozygous *Polr3a*^G672E/G672E^ mice with heterozygous *Polr3b*^+/R103H^ mice and the resulting pups were bred to obtain double mutant mice (*Polr3a*^*G672E/G672E*^*/Polr3b*^*+/R103H*^*,* hereafter referred to as DM mice) (Fig. 3a). DM mice were viable, reproduced normally and did not display a grossly abnormal phenotype. To evaluate balance and coordination, we performed the balance beam test at 6 months of age. We did not detect statistically significant differences in latency to cross the beam or in the number of foot slips between WT and DM mice, suggesting that DM mice do not have impaired gait or cerebellar ataxia (Fig. 3b). Furthermore, WT and DM mice performed comparably in the open field test at 6 months of age, indicating normal general locomotion and voluntary movement (Fig. 3c). We next assessed the presence of hypomyelination and cerebellar atrophy, which are the main pathological features of POLR3-HLD. [5, 9] Coronal brain sections from six month old DM mice stained with an antibody against Proteolipid protein (PLP), one of the main protein components of myelin, were indistinguishable from age-matched WT mice, suggesting that DM mice undergo complete myelination (Fig. 4a). Moreover, Nissl staining and Purkinje cell counts showed normal cerebellar integrity in DM mice (Fig. 4b). Altogether, our behavioral and histological data indicate that DM mice do not recapitulate the phenotypic or pathological features of POLR3-HLD at 6 months of age, and thus that the heterozygous *Polr3b* c.308G > A mutation does not lead to a phenotype in *Polr3a*^G672E/G672E^ mice. Lastly, we evaluated the transcriptional impact of *Polr3a* and *Polr3b* mutations in the brain of DM mice. Using RNA-sequencing (RNA-seq), we found that *Polr3a* and *Polr3b* mRNA levels were comparable in WT and DM mice (Fig. 5a), providing further evidence that the two missense mutations do not alter mRNA stability. Furthermore, the three Pol III transcripts detected by this technique (*Rpph1*, *Rmrp* and *Rn7sk*) appeared unaffected by the *Polr3a* and *Polr3b* mutations (Fig. 5b). In addition, no protein-coding genes showed significant differences (adjusted *p*-value < 0.05 and absolute log2 fold change > 1) between the two groups, demonstrating that *Polr3a* G672E and *Polr3b* R103H missense mutations do not have a global impact on the brain transcriptome. We also measured the levels of one precursor tRNA and one mature tRNA by Northern Blot and found no change in their amounts in WT and DM mice (Fig. 5c), suggesting that 50% wild-type *Polr3b* is sufficient to maintain Pol III function. Thus, our findings strongly suggest that adding a heterozygous mutation in a second Pol III subunit is insufficient to impair mouse neurological function or Pol III transcript levels in *Polr3a*^G672E/G672E^ mice at six months of age.

Characterization of *Polr3a*^G672E/G672E^, *Polr3b*^*R103H/R103H*^ and DM mice clearly shows that the impact of Pol III mutations differs in human and mice. Whether this is due to inter-species differences in myelination, to variable Pol III function or regulation, or to the existence of species-specific Pol III transcripts remains unclear. As evidence for the latter, the most affected Pol III transcript detected in several POLR3-HLD human cellular models, including patient-derived fibroblasts, is BC200 RNA [8], suggesting a role for this primate-specific RNA in POLR3-HLD pathophysiology [10]. BC200 RNA has a functional analog in mouse, Bc1 RNA, which is also synthesized by Pol III. While both transcripts play a similar role in translational regulation, they have different evolutionary origins [10] and recent evidence indicates that BC200 RNA is also involved in mRNA splicing and stability [11, 12]. We found that BC200 RNA was downregulated in patient fibroblasts harboring the POLR3A G672E mutation [8], while Bc1 RNA was not affected in mouse brains homozygous for the same mutation [6], further suggesting that the two transcripts may have different sensitivities to Pol III hypofunction that could explain the absence of neurological phenotype in mice. *Bc1* knockout (KO) in mouse does not lead to hypomyelination or neurological deficits [13]. In contrast, in a human oligodendroglial cell line, both the *POLR3A* M852 V mutation and BC200 RNA KO led to decreased *Myelin basic protein (MBP)* mRNA levels compared to wild-type cells upon cellular differentiation, indicating that Pol III hypofunction could have a detrimental impact on myelin biogenesis and that this effect may be exerted through impaired BC200 RNA expression [8]. Nonetheless, the effect on *MBP* expression was milder in BC200 KO compared to POLR3A-mutated cells, suggesting that multiple factors are at play in the latter and that other Pol III transcripts such as tRNAs, which are conserved in mouse, could contribute to POLR3-HLD.

While it is possible that POLR3-HLD simply cannot be properly modeled in mice, other genetic, developmental or environmental factors that are absent in laboratory mice may be at play in human cases and contribute to pathogenesis. For instance, in addition to its role in nuclear gene transcription, Pol III also acts as a DNA sensor in innate immunity [14]. Since approximately half of POLR3-HLD patients undergo neurological deterioration upon infections, [5] exposure to certain pathogens may play a role in disease progression via this lesser-known function of Pol III.

In conclusion, this study further highlights the difficulty of establishing an appropriate mouse model for POLR3-HLD. Nonetheless, the fact that the *Polr3b*^R103H/R103H^ mice are embryonic lethal while the *Polr3a*^*G672E/G672E*^ mice have no CNS phenotype raises the possibility that other mutations in genes encoding Pol III subunits could lead to intermediate phenotypes that would resemble POLR3-HLD. Furthermore, the possible correlation between mouse phenotype and impact on Pol III function provides a useful tool to select such candidate mutations.

## Materials and methods

### Animals

All experiments were carried out according to good practice of handling laboratory animals consistent with the Canadian Council on Animal Care and approved by the University Animal Care Committee. The mouse line C57BL/6 N-Polr3b < em4Tcp>(*Polr3b*^*+/R103H*^) was made as part of the NorCOMM2 project funded by the Care4Rare Canada Consortium, Genome Canada and the Ontario Genomics Institute (OGI-051) at the Toronto Centre for Phenogenomics. It was obtained from the Canadian Mouse Mutant Repository. Briefly, the mutant allele was created using CRISPR-Cas9, by injecting the Cas9 protein, single guide RNA (5′-TCATGTCCCTCAGACGGCAC-3′) and single-strand oligonucleotide repair template (5′-gtgcactgtattcacagtgggattgctggagggcaggggtcgctcgctcactgtgattctgcttcagTGCCATCTGAGGGACATGACGTACTCCGCCCCAATCACAGTGGACATTGAGTATACCCGAGGCAGCCAGAGGA-3′) including the c.308G > A mutation and the intronic mutation c.304-4C > T to prevent re-editing of the properly mutated genomic DNA. For determination of the lethality of *Polr3b*^*R103H/R103H*^ mice, heterozygous *Polr3b*^*+/R103H*^mice were interbred and gestating females were sacrificed at E9.5 to extract embryos for genotyping. The generation of *Polr3a*^*G672E/G672E*^mice was previously described [6]. For the production of DM mice, *Polr3a*^*G672E/G672E*^ and *Polr3b*^*+/R103H*^ were bred, resulting in 50% *Polr3a*^*+/G672E*^*/Polr3b*^*+/R103H*^ and 50% *Polr3a*^*+/G672E*^*/Polr3b*^*+/+*^ mice. *Polr3a*^*+/G672E*^*/Polr3b*^*+/R103H*^ males and females were subsequently bred together to generate *Polr3a*^*G672E/G672E*^*/Polr3b*^*+/R103H*^ DM mice.

### Genotyping and sanger sequencing

Genomic DNA was extracted from tail biopsies or whole embryos using the Gentra Puragene Tissue Kit (Qiagen). Genotyping of the *Polr3b* R103H allele was performed with two alternative techniques. We first used real-time PCR allelic discrimination and LNA probes (Integrated DNA Technologies) (forward primer: 5′-GCTCGCTCACTGTGATTCT-3′, reverse primer: 5′-CTCGGGTATACTCAATGTCCAC-3′, *Polr3b* WT LNA probe: HEX-CT + CAG + A + C + GGC + AC-IowaBlack FQ, *Polr3b* R103H LNA probe: FAM-AG + A + T + GGCA+CTGG-IowaBlack FQ). We later used PCR amplification of *Polr3b* exon 6 (primers: 5′-GCCAAGCACACACATGTTCTA-3′ and 5′-TACCATTTCCCACACCCTTC-3′) followed by digestion with the restriction enzyme BsrI. The presence of the c.308G > A mutation abolishes the BsrI restriction site. Genotyping of the *Polr3a* G672E allele was conducted as previously reported [6]. For Sanger sequencing of *Polr3b* exon 6, genomic DNA was amplified by PCR using the following primers: 5′-ATTCACAGTGGGATTGCTGG-3′ and 5′-TGGCTTAAGGTCACATCATGG-3′. PCR products were sequenced at the McGill University and Genome Quebec Innovation Center, using a 3730XL DNA Analyzer (Applied Biosystems). Sequence chromatograms were analyzed using SeqMan v.4.03 (DNASTAR Inc., Madison, WI, USA).

### Plasmid construction

POLR3B WT DNA sequence was amplified by PCR using the following primers (Forward 5′-AAGGAAAAAAGCGGCCGCATGGACGTGCTAGCGGAGGAGTT-3′ and Reverse 5′-CTAGTCTAGATTCATTGTACTTGGACAGTTTTAACC-3′) and transferred in a p3XFLAG-CMV-14 expressing vector (Sigma). The c.308G > A (p.R103H) POLR3B mutant was generated by site-directed mutagenesis by amplifying the WT-expressing plasmid with overlapping primers encoding the desired mutation with a Q5 hot-start high-fidelity DNA polymerase (New England Biolabs) and overnight digestion of the parental plasmid with DpnI (New England Biolabs) prior to transformation. All POLR3B plasmid sequences were verified by sequencing.

### Affinity purification coupled with mass spectrometry (AP-MS)

Human embryonic kidney cell line 293 (HEK293) were maintained in culture in DMEM media supplemented with 10% fetal bovine serum and 2 mM glutamine, seeded in 6-wells plates at 3 × 10^5^ cells/well, grown overnight and transiently transfected with 500 ng of FLAG-tagged WT or R103H POLR3B expressing plasmids by using Jet Prime transfection reagent (PolyPlus) according to manufacturer’s instruction. Transfected cells were incubated at 37 °C for 24 h, washed with sterile PBS, pelleted at 3500RPM and snap frozen in liquid nitrogen. Affinity purifications were performed using standard procedures in four independent replicate experiments [3]. The speed-vac protein extracts were then re-solubilized in 10 μL of a 6 M urea buffer, reduced in reduction buffer (45 mM DTT, 100 mM ammonium bicarbonate) for 30 min at 37 °C, and alkylated in alkylation buffer (100 mM iodoacetamide, 100 mM ammonium bicarbonate) for 20 min at 24 °C in dark and proteins were digested in 10 μL of the trypsin solution (5 ng/μL of trypsin sequencing grade from Promega, 50 mM ammonium bicarbonate) at 37 °C for 18 h. The protein digests were acidified with trifluoroacetic acid for desalting and removal of residual detergents by MCX (Waters Oasis MCX 96-well Elution Plate) according to manufacturer’s instructions. The resulting peptides were identified with tandem mass spectrometry (LC-MS/MS) using a HPLC system coupled to an Orbitrap Fusion mass spectrometer (Thermo Scientific) through a Nanospray Flex Ion Source. MS/MS raw data were searched against the Uniprot database (updated on June 12^th^2018) using X-Tandem (version 2013.06.15.1) [15] for protein identification and spectral counts were computed by Prohits (version 6.0.4) [16]. Spectral counts were transferred in Perseus (Version 1.6.1.3) [17]. Proteins quantified in three out of four experiments for either WT or R103H POLR3B were kept for further analysis. Spectral counts reported as 0 by X-Tandem were replaced by a randomly generated spectral count value normally distributed with a mean and S.D. equal to those of the lowest 20% spectral count values from the LC-MS/MS analysis. Spectral counts were normalized by the spectral count of the bait (POLR3B) in order to allow comparison between different purifications. WT and R103H POLR3B proteins were compared to the FLAG empty vector control samples and were labeled as high-confidence interactors when their *p*-value was under 0.05 and their spectral count ratio was over 2. Statistical differences between WT and R103H POLR3B were determined using a two-tailed T-test subsequently adjusted for multiple hypothesis testing using a permutation-based test by considering a False discovery rate (FDR) of 5% adjusted using an s0 correction factor of 0.1 with 10,000 iterations [18]. The level of differential interaction with POLR3B were considered statistically significant when the adjusted *P* value was < 0.05 and its average spectral count fold-change (R103H/WT) was ±2.

### Behavioral tests

Six months old WT and DM mice were submitted to the balance beam, rotarod and open field tests as previously described [6]. Male (5 WT, 3 DM) and female (3 WT, 5 DM) mice were used for the balance beam and rotarod tests, while an independent cohort of males (7 WT, 8 DM) were subsequently used for the open field test.

### Histology

For preparation of tissue sections, mouse cerebrum and cerebellum were harvested and fixed in 4% paraformaldehyde for 24 h at 4 °C. Tissues were then equilibrated in 30% sucrose/PBS for 48 h. Sagittal or coronal sections (30 μm) were cut using a freezing sledge microtome. Free-floating sections were processed for immunofluorescence as previously described [19]. Sections were labeled with an antibody against PLP (Abcam #ab28486, 1/200) in three mice per group. Nissl stains and Purkinje cell counts were performed in two mice per group as previously described [20]. Imaging was performed using a Zeiss Axiovert M2 microscope.

### RNA extraction, RT-PCR and Northern blots

Cerebral and cerebellar hemispheres were harvested and snap-frozen in liquid nitrogen. Tissues were homogenized in Qiazol lysis reagent (Qiagen). Total RNA was extracted with the miRNeasy kit (Qiagen) and treated with DNAse I (Qiagen) according to the manufacturer’s instructions. For RT-PCR of *Polr3b *exons 4–8, brain RNA was reversed transcribed into complementary DNA (cDNA) using the Superscript III Reverse Transcriptase (ThermoFisher) according to the manufacturer’s instructions and amplified by PCR using the following primers: 5′-CAGCACATAGACTCCTTTAACTATTTC-3’and 5′-GGGTGGAGCTGGTAACTGA-3′. Northern Blots for precursor and mature tRNAs were performed as previously described [6].

### RNA-sequencing

Illumina TruSeq rRNA-depleted stranded libraries were prepared from total cerebrum RNA of 6 months old male mice (3 WT, 3 DM) and sequenced on an Illumina HiSeq 2500 with 125 bp paired-end reads at an average of 38.7 million reads/sample. Quality control and trimming were performed as previously described [21]. Trimmed reads were aligned to the reference genome mm10 using STAR v2.3.0e [22]. Expression levels were estimated with featureCounts [23] using exonic reads and normalized using DESeq2 [24]. Differential expression analysis was performed with DESeq2.

## Additional files

Additional file 1: **Figure S1.** FLAG-POLR3B R103H and FLAG-POLR3B WT expression levels. (PDF 2856 kb)

Additional file 2: **Table S1.** AP-MS data for POLR3B WT and R103H (XLSX 22 kb)

## Data Availability

The datasets used and/or analysed during the current study are available from the corresponding author on reasonable request.

## Abbreviations

AP-MS: Affinity purification coupled to mass spectrometry
cDNA: Complementary DNA
CNS: Central nervous system
DM: Double mutant mice
HLD: Hypomyelinating leukodystrophy
MRI: Magnetic resonance imaging
ncRNA: Non-coding RNA
PLP: Proteolipid protein
Pol III: RNA Polymerase III
rRNA: Ribosomal RNA
SEM: Standard error of the mean
tRNA: Transfer RNA
WT: Wild-type

## References

[1] Bernard G, Chouery E, Putorti ML, Tetreault M, Takanohashi A, Carosso G, Clement I, Boespflug-Tanguy O, Rodriguez D, Delague V (2011). Mutations of POLR3A encoding a catalytic subunit of RNA polymerase pol III cause a recessive hypomyelinating leukodystrophy. Am J Hum Genet.

[2] Tetreault M, Choquet K, Orcesi S, Tonduti D, Balottin U, Teichmann M, Fribourg S, Schiffmann R, Brais B, Vanderver A, Bernard G (2011). Recessive mutations in POLR3B, encoding the second largest subunit of pol III, cause a rare hypomyelinating leukodystrophy. Am J Hum Genet.

[3] Thiffault I, Wolf NI, Forget D, Guerrero K, Tran LT, Choquet K, Lavallee-Adam M, Poitras C, Brais B, Yoon G (2015). Recessive mutations in POLR1C cause a leukodystrophy by impairing biogenesis of RNA polymerase III. Nat Commun.

[4] Dorboz I, Dumay-Odelot H, Boussaid K, Bouyacoub Y, Barreau P, Samaan S, Jmel H, Eymard-Pierre E, Cances C, Bar C (2018). Mutation in POLR3K causes hypomyelinating leukodystrophy and abnormal ribosomal RNA regulation. Neurol Genet.

[5] Wolf NI, Vanderver A, van Spaendonk RM, Schiffmann R, Brais B, Bugiani M, Sistermans E, Catsman-Berrevoets C, Kros JM, Pinto PS (2014). Clinical spectrum of 4H leukodystrophy caused by POLR3A and POLR3B mutations. Neurology.

[6] Choquet K, Yang S, Moir RD, Forget D, Lariviere R, Bouchard A, Poitras C, Sgarioto N, Dicaire MJ, Noohi F (2017). Absence of neurological abnormalities in mice homozygous for the Polr3a G672E hypomyelinating leukodystrophy mutation. Mol Brain.

[7] Sievers F, Wilm A, Dineen D, Gibson TJ, Karplus K, Li W, Lopez R, McWilliam H, Remmert M, Soding J (2011). Fast, scalable generation of high-quality protein multiple sequence alignments using Clustal omega. Mol Syst Biol.

[8] Choquet K, Forget D, Meloche E, Dicaire MJ, Bernard G, Vanderver A, Schiffmann R, Fabian MR, Teichmann M, Coulombe B (2019). Leukodystrophy-associated POLR3A mutations down-regulate the RNA polymerase III transcript and important regulatory RNA BC200. J Biol Chem.

[9] Vanderver A, Tonduti D, Bernard G, Lai J, Rossi C, Carosso G, Quezado M, Wong K, Schiffmann R (2013). More than hypomyelination in pol-III disorder. J Neuropathol Exp Neurol.

[10] Tiedge H, Chen W, Brosius J (1993). Primary structure, neural-specific expression, and dendritic location of human BC200 RNA. J Neurosci.

[11] Shin H, Lee J, Kim Y, Jang S, Lee Y, Kim S, Lee Y (2017). Knockdown of BC200 RNA expression reduces cell migration and invasion by destabilizing mRNA for calcium-binding protein S100A11. RNA Biol.

[12] Singh R, Gupta SC, Peng WX, Zhou N, Pochampally R, Atfi A, Watabe K, Lu Z, Mo YY (2016). Regulation of alternative splicing of Bcl-x by BC200 contributes to breast cancer pathogenesis. Cell Death Dis.

[13] Skryabin BV, Sukonina V, Jordan U, Lewejohann L, Sachser N, Muslimov I, Tiedge H, Brosius J (2003). Neuronal untranslated BC1 RNA: targeted gene elimination in mice. Mol Cell Biol.

[14] Chiu YH, Macmillan JB, Chen ZJ (2009). RNA polymerase III detects cytosolic DNA and induces type I interferons through the RIG-I pathway. Cell.

[15] Craig R, Beavis RC (2004). TANDEM: matching proteins with tandem mass spectra. Bioinformatics.

[16] Liu G, Zhang J, Larsen B, Stark C, Breitkreutz A, Lin ZY, Breitkreutz BJ, Ding Y, Colwill K, Pasculescu A (2010). ProHits: integrated software for mass spectrometry-based interaction proteomics. Nat Biotechnol.

[17] Tyanova S, Temu T, Sinitcyn P, Carlson A, Hein MY, Geiger T, Mann M, Cox J (2016). The Perseus computational platform for comprehensive analysis of (prote)omics data. Nat Methods.

[18] Tusher VG, Tibshirani R, Chu G (2001). Significance analysis of microarrays applied to the ionizing radiation response. Proc Natl Acad Sci U S A.

[19] Lariviere R, Gaudet R, Gentil BJ, Girard M, Conte TC, Minotti S, Leclerc-Desaulniers K, Gehring K, McKinney RA, Shoubridge EA (2015). Sacs knockout mice present pathophysiological defects underlying autosomal recessive spastic ataxia of Charlevoix-Saguenay. Hum Mol Genet.

[20] Girard M, Lariviere R, Parfitt DA, Deane EC, Gaudet R, Nossova N, Blondeau F, Prenosil G, Vermeulen EG, Duchen MR (2012). Mitochondrial dysfunction and Purkinje cell loss in autosomal recessive spastic ataxia of Charlevoix-Saguenay (ARSACS). Proc Natl Acad Sci U S A.

[21] Antonicka H, Choquet K, Lin ZY, Gingras AC, Kleinman CL, Shoubridge EA (2017). A pseudouridine synthase module is essential for mitochondrial protein synthesis and cell viability. EMBO Rep.

[22] Dobin A, Davis CA, Schlesinger F, Drenkow J, Zaleski C, Jha S, Batut P, Chaisson M, Gingeras TR (2013). STAR: ultrafast universal RNA-seq aligner. Bioinformatics.

[23] Liao Y, Smyth GK, Shi W (2014). featureCounts: an efficient general purpose program for assigning sequence reads to genomic features. Bioinformatics.

[24] Love MI, Huber W, Anders S (2014). Moderated estimation of fold change and dispersion for RNA-seq data with DESeq2. Genome Biol.

