# Supplementary material for: The leukodystrophy mutation Polr3b R103H causes homozygote mouse embryonic lethality and impairs RNA polymerase III biogenesis
Source: Mol Brain. 2019 Jun 20;12:59. doi: 10.1186/s13041-019-0479-7 (PMC6587292; doi:10.1186/s13041-019-0479-7)
Supplement: Supplementary file 1 — Figure S1. FLAG-POLR3B R103H and FLAG-POLR3B WT expression levels. (PDF 2856 kb) [file 13041_2019_479_MOESM1_ESM.pdf]

Supplementary Figure 1

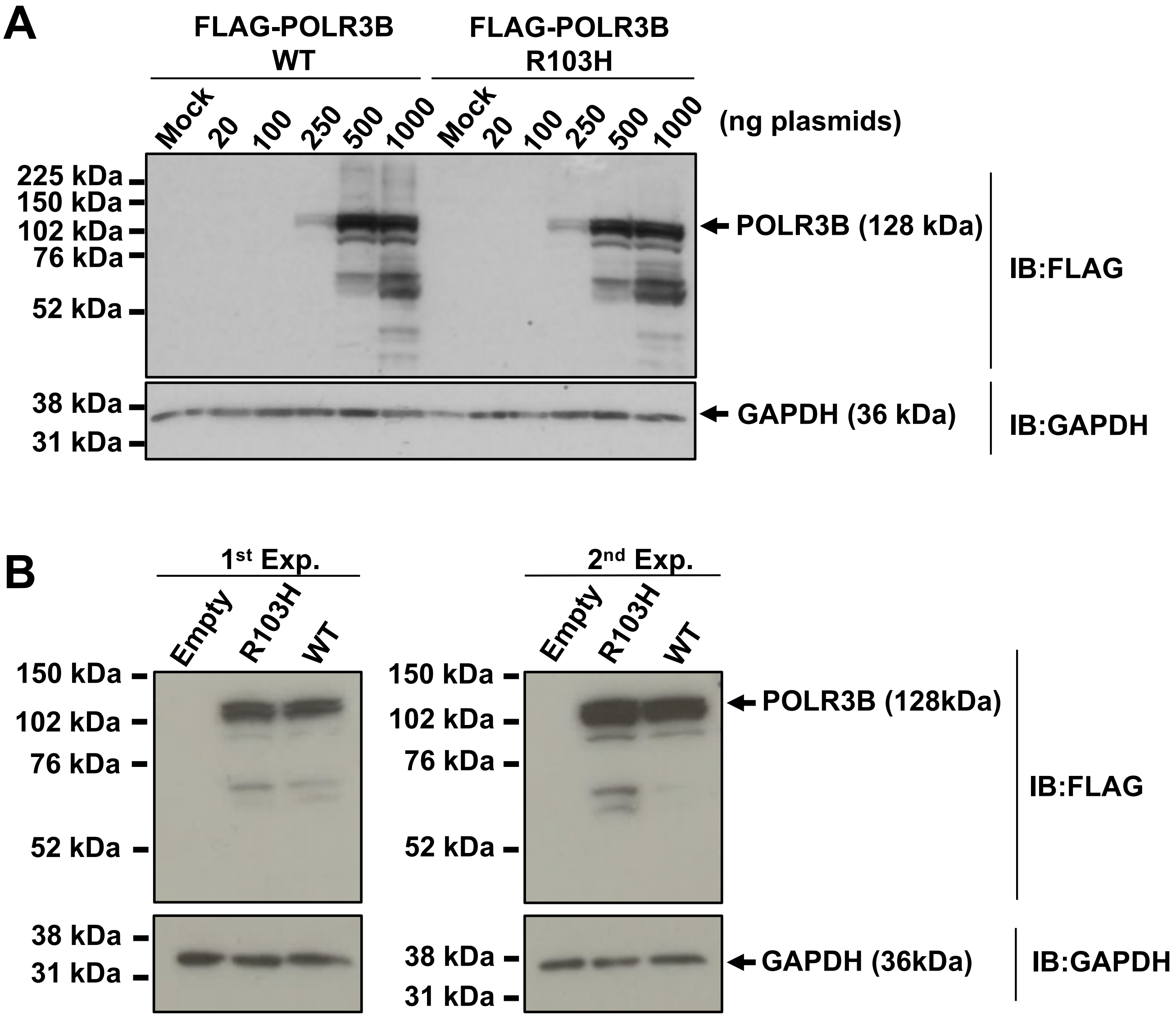

Supplementary Figure 1. FLAG-POLR3B R103H expression level is similar to FLAG-POLR3B WT. A) Protein expression of the FLAG-POLR3B WT or the R013H mutant was assessed by immunoblot following transfection of various quantity of plasmid in HEK 293S as indicated and B) 500ng of either FLAG-POLR3B WT, or R013H mutant, or FLAG-empty vector expressing plasmid was transfected in HEK293S in two independent experiments. 30μg of whole cell protein extracts were loaded and FLAG-POLR3B WT or R103H were detected using anti-FLAG (M2) antibody. GAPDH was used as a loading control. IB: immunoblot; WT: wild-type; Empty: FLAG-empty vector negative control.
